# Heart Size Difference Drives Sex‐Specific Response to Cardiac Resynchronization Therapy: A Post Hoc Analysis of the MORE‐MPP CRT Trial

**DOI:** 10.1161/JAHA.123.035279

**Published:** 2024-06-15

**Authors:** Nadeev Wijesuriya, Vishal Mehta, Felicity De Vere, Sandra Howell, Steven A. Niederer, Haran Burri, Johannes Sperzel, Leonardo Calo, Bernard Thibault, Wenjiao Lin, Kwangdeok Lee, Andrea Grammatico, Niraj Varma, Marianne Gwechenberger, Christophe Leclercq, Christopher A. Rinaldi

**Affiliations:** ^1^ King’s College London London UK; ^2^ Guy’s and St Thomas’s NHS Foundation Trust London UK; ^3^ National Heart and Lung Institute Imperial College London London UK; ^4^ University Hospital of Geneva Geneva Switzerland; ^5^ Kerckhoff—Klinik Heart Center Bad Nauheim Germany; ^6^ Casilino Policlinico Rome Italy; ^7^ Montreal Heart Institute Montreal QC Canada; ^8^ Abbott Plano TX USA; ^9^ Cleveland Clinic London UK; ^10^ Medical University of Vienna Vienna Austria; ^11^ CHRU Pontchaillou Rennes France

**Keywords:** cardiac resynchronization therapy, heart failure, sex differences, Heart Failure

## Abstract

**Background:**

Studies have reported that female sex predicts superior cardiac resynchronization therapy (CRT) response. One theory is that this association is related to smaller female heart size, thus increased relative dyssynchrony at a given QRS duration (QRSd). Our objective was to investigate the mechanisms of sex‐specific CRT response relating to heart size, relative dyssynchrony, cardiomyopathy type, QRS morphology, and other patient characteristics.

**Methods and Results:**

This is a post hoc analysis of the MORE‐CRT MPP (More Response on Cardiac Resynchronization Therapy with Multipoint Pacing)  trial (n=3739, 28% women), with a subgroup analysis of patients with nonischemic cardiomyopathy and left bundle‐branch block (n=1308, 41% women) to control for confounding characteristics. A multivariable analysis examined predictors of response to 6 months of conventional CRT, including sex and relative dyssynchrony, measured by QRSd/left ventricular end‐diastolic volume (LVEDV). Women had a higher CRT response rate than men (70.1% versus 56.8%, *P*<0.0001). In subgroup analysis, regression analysis of the nonischemic cardiomyopathy left bundle‐branch block subgroup identified QRSd/LVEDV, but not sex, as a modifier of CRT response (*P*<0.0039). QRSd/LVEDV was significantly higher in women (0.919) versus men (0.708, *P*<0.001). CRT response was 78% for female patients with QRSd/LVEDV greater than the median value, compared with 68% with QRSd/LVEDV less than the median value (*P*=0.012). The association between CRT response and QRSd/LVEDV was strongest at QRSd <150 ms.

**Conclusions:**

In the nonischemic cardiomyopathy left bundle‐branch block population, increased relative dyssynchrony in women, who have smaller heart sizes than their male counterparts, is a driver of sex‐specific CRT response, particularly at QRSd <150 ms. Women may benefit from CRT at a QRSd <130 ms, opening the debate on whether sex‐specific QRSd cutoffs or QRS/LVEDV measurement should be incorporated into clinical guidelines.

**Nonstandard Abbreviations and Acronyms**
NICM: nonischemic cardiomyopathy

**Clinical Perspective**

**What Is New?**

- In patients with nonischemic cardiomyopathy and left bundle‐branch block, increased normalized QRS duration/left ventricular end diastolic volume in those with smaller heart sizes drives the improved cardiac resynchronization therapy response rates observed in women compared with men.
- This association is strongest at QRS durations <150 ms.

**What Are the Clinical Implications?**

- There may be a cohort of female patients with QRS durations <130 ms, but a high degree of relative dyssynchrony, who could benefit from cardiac resynchronization therapy despite falling outside current guidelines. This opens the debate on whether normalized QRS duration or sex‐specific QRS duration cutoffs have a place in clinical practice.

Cardiac resynchronization therapy (CRT) is a hallmark treatment for patients with dyssynchronous heart failure.^1^ However, despite its widespread success and uptake, 30% of patients fail to derive benefit.^2^, ^3^ These CRT nonresponders have among the poorest long‐term outcomes of any subgroup in the heart failure population.^4^ As such, there is significant interest in examining factors that modulate CRT response.

It has been well established that certain conditions are associated with a poorer clinical or left ventricular (LV) remodeling responses to CRT, for example, ischemic cardiomyopathy, atrial fibrillation (AF), and non‐left bundle‐branch block (LBBB) QRS morphology.^5^ These associations are unsurprising from a physiological perspective. An area that remains poorly understood is the association of male or female sex in CRT response. Although some studies report no sex differences, for example CARE‐HF (Cardiac Resynchronization in Heart Failure),^6^ most have reported that female sex is predictive of superior clinical and echocardiographic response.^7^, ^8^, ^9^, ^10^, ^11^, ^12^ In a meta‐analysis of 149 259 patients, Yin et al observed a lower all‐cause mortality in women than men post‐CRT (odds ratio [OR], 0.50 [95% CI, 0.36–0.70]).^13^ Women also exhibited statistically significant improvement in LV ejection fraction and decrement of LV end‐diastolic diameter when compared with men.

The mechanisms underpinning this association remain unclear. One theory is that the higher female response rate is related to a higher frequency of LBBB and nonischemic cardiomyopathy (NICM) phenotypes. Determining this accurately requires examination of large sample sizes, especially because women represent only 20% to 30% of the population in CRT trials.^14^

Sex‐related CRT response disparity may also be explained by differences in cardiac size,^15^ that is, that women are relatively smaller than men, and therefore have a greater degree of dyssynchrony at a given QRS duration (QRSd). This is supported by studies that report that women exhibit an improved CRT response compared with men in cohorts with a QRSd <150 ms.^16^, ^17^, ^18^ It is suggested that sex‐specific differences in QRS response relationship are unexplained by the application of strict LBBB criteria or by body surface area, but resolved by QRSd normalization for heart size using LV mass or LV end‐diastolic volume (LVEDV).^19^, ^20^ These metrics of relative dyssynchrony, such as QRSd/LVEDV, are not routinely measured in clinical practice, nor are they frequently reported in larger CRT trials. As such, this area has, to date, only been examined in single‐center cohorts with small sample sizes. A meta‐analysis of 3496 patients identified height and QRS duration, but not sex, as independent predictors for the composite outcome of first hospitalization for heart failure and all‐cause mortality; however, QRSd/LVEDV was not used in this analysis.^21^

In this study, we aimed to determine the association between sex, LV size, and relative dyssynchrony in a large cohort of CRT recipients. We tested this by performing a subanalysis of patients recruited to the MORE‐CRT MPP (More Response on Cardiac Resynchronization Therapy with Multipoint Pacing)  trial.

## Methods

MORE‐CRT MPP is a prospective, randomized multicenter study.^22^, ^23^ All patients initially received conventional biventricular (BiV) CRT for 6 months. At this stage, echocardiographic nonresponders are randomized to either continued conventional BiV pacing or multipoint pacing. CRT response was defined as a reduction in LV end‐systolic volume >15% analyzed by an independent core laboratory. We evaluated data following the first 6 months of conventional BiV CRT; a post hoc multivariable analysis examined predictors of this reverse remodeling response (Figure S1). In particular, we assessed the predicted probability of CRT response according to sex and QRSd/LVEDV. Given the significant confounding effects on a regression analysis of known highly predictive variables such as QRS morphology and cardiomyopathy phenotype, a subgroup analysis of the NICM LBBB population was performed to test the effects of sex differences and normalized QRSd in a less heterogenous population. The data that support the findings of this study are available from the corresponding author upon reasonable request. The MORE‐CRT MPP study was approved by an institutional review board, and all subjects gave informed consent. A full list of participating principal investigators and institutions can be found in Table S1.

### Study Participants

The study enrolled eligible patients with a standard CRT indication after obtaining written informed consent. The device was programmed BiV pacing in those patients who had the quadripolar CRT system successfully implanted (Quartet LV lead with a Quadra CRT device; Abbott, Sylmar, CA) with programming of the LV pacing vector, atrioventricular  delay, and interventricular  delay settings at the implanting physician's discretion. During the first 6 months following implant, all subjects received standard BiV pacing.

### Echocardiographic Assessment

Trained and qualified site personnel performed echocardiographic recordings, with analysis performed by an independent echocardiography core laboratory. Simpson biplane method using end‐diastolic and end‐systolic volumes obtained in the apical 2‐chamber and 4‐chamber views were used to measure left ventricular ejection fraction. All measurements were made at baseline and at 6 months.

### Statistical Analysis

Summary statistics were used for baseline characteristics. Continuous variables were summarized by mean±SD. Two‐sample Student *t* test or Wilcoxon rank sum test was used to test the difference between 2 groups depending on the normality of the data. Frequencies and percentages summarized categorical variables. A χ^2^ test or Fisher exact test was used to test the difference between groups.

We performed univariable models using the following baseline variables: age, AF, chronic obstructive pulmonary disease, diabetes, hypercholesterolemia, hypertension, LVEDV, left ventricular ejection fraction, New York Heart Association Class I/II versus Class III/IV, renal disease, ischemic versus NICM, LBBB versus non‐LBBB, QRSd, QRSd/LVEDV (normalized QRS), and sex. Multivariable models were conducted using stepwise selections. The criteria for baseline variables entering into and staying in the model were α=0.25 and α=0.05, respectively. Age and sex were forced into the model. Given that logistic regression methods rely on the assumption of linearity between CRT response and the predicting independent variables (patients’ characteristics), we also evaluated the probability of CRT response as a function of QRSd (divided in 5 subgroups) and as a function of QRSd normalized by dividing it by LVEDV (divided in 10 deciles) to account for heart size differences between women and men. The predicted probability of CRT response as a function of QRSd and normalized QRSd was derived from multivariable logistic regression models. Analyses were conducted using SAS 9.4.

## Results

### Baseline Characteristics

Only patients with left ventricular end‐systolic volume data available at baseline and 6 months were included in this analysis. Of 3906 patients who completed the 6‐month follow‐up, analysis was performed on 3739 patients (1051, 28% women). Patients’ baseline characteristics are shown in Tables 1 and 2. In the whole population (Table 1), the LVEDV in women was significantly smaller compared with men (176±60 mL versus 228 ±77 mL, *P*<0.0001). Women had a significantly shorter QRSd than men (153±22 ms versus 158±26 ms, *P*<0.0001). Similar results on cardiac size and QRSd were observed in the nonischemic LBBB subgroup (Table 2).

**Table 1:** Baseline Patient Characteristics for the Whole Population

Demographic variable	All subjects (n=3739)	Female subjects (n=1051)	Male subjects (n=2688)	*P* value
Age (y)				
Mean±SD	68±11	68±11	68±11	0.3454*
NYHA class at enrollment (%)				
Class II	51.2	45.3	53.5	
Class III	46.6	52.1	44.5	<0.0001†
Class IV	1.9	2.4	1.7	
QRS duration (ms)				
Mean±SD	156±25	153±22	158±26	<0.0001*
QRS morphology (%)				
LBBB	70.2	79.5	66.4	<0.0001†
Non‐LBBB	29.8	20.5	33.6	
Cardiomyopathy cause (%)				
Ischemic	41.0	22.0	48.4	<0.0001†
Nonischemic	59.0	78.0	51.6	
LVESV (mL)				
Mean±SD	160±65	131±52	171±66	<0.0001*
LVEDV (mL)				
Mean±SD	214±76	176±60	228±77	<0.0001*
LVEF (%)				
Mean±SD	26±7	27±7	26±7	0.0965*
Device type (%)				
CRT‐P	12.2	16.9	10.4	<0.0001†
CRT‐D	87.8	83.1	89.6	
Medical history (%)				
Hypertension	62.3	59.0	63.6	0.0087†
Hypercholesterolemia	40.1	34.6	42.4	<0.0001†
Diabetes	33.4	30.4	34.6	0.0126†
COPD	10.6	8.5	11.5	0.0076†
Renal disease	15.0	11.4	16.4	0.0001†
Medical treatment (%)				
Diuretics	77.4	79.5	76.5	0.0474†
ACE inhibitor/ARB	89.3	89.4	89.2	0.8400†
β‐Blocker	89.1	89.2	89.1	0.8697†
Aldosterone antagonist	38.9	38.5	39.1	0.7660†
Anticoagulant	26.9	21.4	29.1	<0.0001†
Calcium channel blocker	7.9	6.2	8.6	0.0156†
Nitrates	7.4	5.6	8.1	0.0097†

ACE indicates angiotensin‐converting enzyme; ARB, angiotensin receptor blocker; COPD, chronic obstructive pulmonary disease; CRT‐D, cardiac resynchronization therapy with defibrillator; CRT‐P, cardiac resynchronization therapy without defibrillator; LBBB, left bundle‐branch block; LVEDV, left ventricular end‐diastolic volume; LVEF, left ventricular ejection fraction; LVESV, left ventricular end‐systolic volume; and NYHA, New York Heart Association.

* Wilcoxon rank sum test.

† Pearson χ^2^ test.

**Table 2:** Baseline Patient Characteristics for Patients With Nonischemic Left Bundle‐Branch Block

Demographic variable	All subjects (n=1308)	Female subjects (n=538)	Male subjects (n=770)	*P* value
Age (y)				
Mean±SD	66±11	67±10	65±11	0.0039*
NYHA class at enrollment (%)				
Class II	55.4	45.5	62.2	<0.0001†
Class III	42.2	51.5	35.7	
Class IV	2.1	2.6	1.8	
QRS duration (ms)				
Mean±SD	161±19	157±18	165±20	<0.0001*
LVESV (mL)				
Mean±SD	162±70	135±54	181±73	<0.0001*
LVEDV (mL)				
Mean±SD	216±82	180±64	241±85	<0.0001*
LVEF (%)				
Mean±SD	26±7	26±7	26±7	0.3278*
Device type (%)				
CRT‐P	11.3	13.9	9.5	0.0122†
CRT‐D	88.7	86.1	90.5	
Medical history (%)				
Hypertension	56.4	55.0	57.4	0.3922†
Hypercholesterolemia	33.1	29.9	35.3	0.0412†
Diabetes	28.4	29.4	27.8	0.5342†
COPD	9.7	8.9	10.3	0.4213†
Renal disease	9.2	9.1	9.2	0.9445†
Medical treatment (%)				
Diuretics	76.6	78.8	75.1	0.1154†
ACE inhibitor/ARB	91.5	90.7	92.1	0.3811†
β‐Blocker	91.1	90.3	91.6	0.4454†
Aldosterone antagonist	40.0	38.3	41.2	0.2956†
Anticoagulant	21.0	17.7	23.4	0.0125†
Calcium channel blocker	5.0	5.2	4.9	0.8266†
Nitrates	3.7	3.5	3.8	0.8242†

ACE indicates angiotensin‐converting enzyme; ARB, angiotensin receptor blocker; COPD, chronic obstructive pulmonary disease; CRT‐D, cardiac resynchronization therapy with defibrillator; CRT‐P, cardiac resynchronization therapy without defibrillator; LVEDV, left ventricular end‐diastolic volume; LVEF, left ventricular ejection fraction; LVESV, left ventricular end‐systolic volume; and NYHA, New York Heart Association.

* Wilcoxon rank sum test.

^†^ Pearson χ^2^ test.

### CRT Response in the Whole Study Cohort

CRT response by sex is outlined in Table 3. Female patients had a higher CRT response rate than men in the total population (70.1% versus 56.8%, *P*<0.0001). The results of the logistic regression model (Table 4) show that LBBB, wide QRSd, and female sex are significant independent predictors for improved response, whereas history of AF, ischemic cause, large heart (LVEDV), and history of renal disease are significant independent predictors for reduced CRT response. When QRSd/LVEDV ratio instead of QRSd was included in a separate model, QRSd/LVEDV was not a significant independent predictor of response (OR, 1.21 [95% CI, 0.89–1.63]; *P*=0.22). There was a significant correlation between LVEDV and QRSd/LVEDV (correlation coefficient −0.794, *P*<0.0001).

**Table 3:** Cardiac Resynchronization Therapy Response at 6 Month Follow Up (6M Responder Rate) by sex

Population	Sex	6M responder rate (n/N)	*P* value
Whole population	Female	70.12% (737/1051)	<0.0001
	Male	56.85% (1528/2688)	
Patients with LBBB	Female	71.07% (479/674)	<0.0001
	Male	62.06% (854/1376)	
Nonischemic patients with LBBB	Female	73.05% (393/538)	0.2508
	Male	70.13% (540/770)	
Nonischemic LBBB and QRSd <150 ms	Female	69.71% (122/175)	0.0157
	Male	56.86% (87/153)	
Patients with non‐LBBB	Female	62.64% (109/174)	<0.0001
	Male	40.29% (280/695)	

LBBB indicates left bundle‐branch block; and QRSd, QRS duration.

**Table 4:** Logistic Regression Results (Whole Population)

Parameter	Univariate			Multivariate (2866 patients)	
	Parameter estimate [95% CI]	*P* value	Sample size	Parameter estimate [95% CI]	*P* value
Age	0.998 [0.992–1.004]	0.5685	3738	1.002 [0.994–1.010]	0.6972
Atrial fibrillation	0.679 [0.565–0.815]	<0.0001	3739	0.785 [0.627–0.982]	0.0339
COPD	1.018 [0.822–1.260]	0.8702	3739		
Diabetes	0.770 [0.671–0.884]	0.0002	3739		
Hypercholesterolemia	0.813 [0.712–0.929]	0.0024	3739		
Hypertension	1.047 [0.914–1.198]	0.5096	3739		
Ischemic vs nonischemic	0.502 [0.439–0.574]	<0.0001	3739	0.550 [0.466–0.648]	<0.0001
LBBB vs non‐LBBB	2.294 [1.952–2.696]	<0.0001	2919	1.911 [1.593–2.291]	<0.0001
LVEDV	0.999 [0.998–1.000]	0.0024	3739	0.998 [0.997–0.999]	0.0035
LVEF	0.996 [0.987–1.005]	0.4062	3739		
NYHA I/II vs III/IV	1.267 [1.111–1.445]	0.0004	3728		
QRSd	1.007 [1.005–1.010]	<0.0001	3223	1.005 [1.001–1.008]	0.0107
Renal disease	0.569 [0.475–0.682]	<0.0001	3739	0.663 [0.532–0.826]	0.0002
Women vs men	1.782 [1.530–2.075]	<0.0001	3739	1.385 [1.144–1.675]	0.0008

COPD indicates chronic obstructive pulmonary disease; LBBB, left bundle‐branch block; LVEDV, left ventricular end‐diastolic volume; LVEF, left ventricular ejection fraction; and NYHA, New York Heart Association; and QRSd, QRS duration.

### CRT Response in the NICM LBBB Cohort

**Figure 1:**
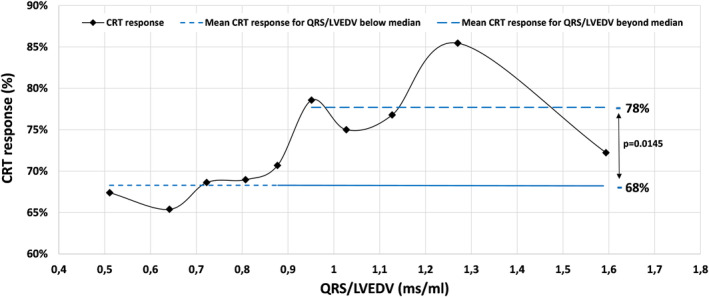
CRT response as a function of QRSd/LVEDV in female patients with NICM LBBB. QRSd/LVEDV was treated as a continuous variable and divided into 10 deciles. CRT, indicates cardiac resynchronization therapy; LBBB, left bundle‐branch block; LVEDV, left ventricular end‐diastolic volume; NICM, nonischemic cardiomyopathy; and QRSd, QRS duration.

In a subgroup analysis of only patients with a NICM LBBB phenotype (1308 patients, 538 women), there was no significant difference in CRT response between men and women (73% versus 70%, *P*=0.25). A logistic regression model (Table 5) when applied to patients with NICM LBBB showed that CRT response was significantly and independently associated with QRSd/LVEDV but not with sex. AF and diabetes were also significant negative response predictors in this analysis. A sensitivity analysis was performed to determine the strength of normalized QRSd association with response. A multivariate model was run that excluded AF and diabetes. This demonstrated only a minimal change in the QRSd/LVEDV OR, suggesting that there was no confounding effect between QRSd/LVEDV and AF/diabetes (Table S2). In this cohort, there was a significant correlation between LVEDV and normalized QRS (correlation coefficient −0.84, *P*<0.0001). Normalized QRS was significantly different between women, who had median value of 0.919 (0.734–1.127) ms/mL, and men, who had median value of 0.708 (0.585–0.886) ms/mL (*P*<0.001). For female patients with a QRSd/LVEDV of >0.915 ms/mL (the median value), the CRT response rate was 78%, compared with 68% in patients where the QRSd/LVEDV was below the median value (*P*=0.012, Figure 1). CRT response as a function of QRSd/LVEDV for men is shown in Figure S2.

**Table 5:** Logistic Regression Results (Nonischemic Left Bundle‐Branch Block Population)

Parameters	Univariate			Multivariate (1295 patients)	
	Parameter estimate [95% CI]	*P* value	Sample size	Parameter estimate [95% CI]	*P* value
Age	1.002 [0.991–1.013]	0.7481	1308	1.000 [0.988–1.012]	0.9983
AF vs no AF	0.651 [0.449–0.945]	0.0240	1308	0.627 [0.430–0.913]	0.0150
COPD yes vs no	1.400 [0.908–2.158]	0.1275	1308		…
Diabetes yes vs no	0.759 [0.585–0.985]	0.0378	1308	0.751 [0.577–0.978]	0.0333
Hypercholesterolemia	0.922 [0.715–1.187]	0.5278	1308		…
Hypertension yes vs no	0.994 [0.780–1.265]	0.9590	1308		…
LVEDV	0.998 [0.997–0.999]	0.0041	1308		…
LVEF	1.009 [0.991–1.026]	0.3307	1308		…
NYHA I/II vs NYHA III/IV	1.115 [0.876–1.419]	0.3751	1304		…
QRSd	1.010 [1.004–1.016]	0.0022	1295		…
QRSd/LVEDV	1.948 [1.267–2.996]	0.0024	1295	1.984 [1.246–3.157]	0.0039
Renal disease yes vs no	0.616 [0.417–0.909]	0.0147	1308		…
Women vs men	1.154 [0.903–1.475]	0.2509	1308	0.987 [0.758–1.285]	0.9202

AF indicates atrial fibrillation; COPD, chronic obstructive pulmonary disease; LVEDV, left ventricular end‐diastolic volume; LVEF, left ventricular ejection fraction; NYHA, New York Heart Association; and QRSd, QRS duration.

### Analysis of Sex‐Impact as a Function of QRS in the NICM LBBB Cohort

**Figure 2:**
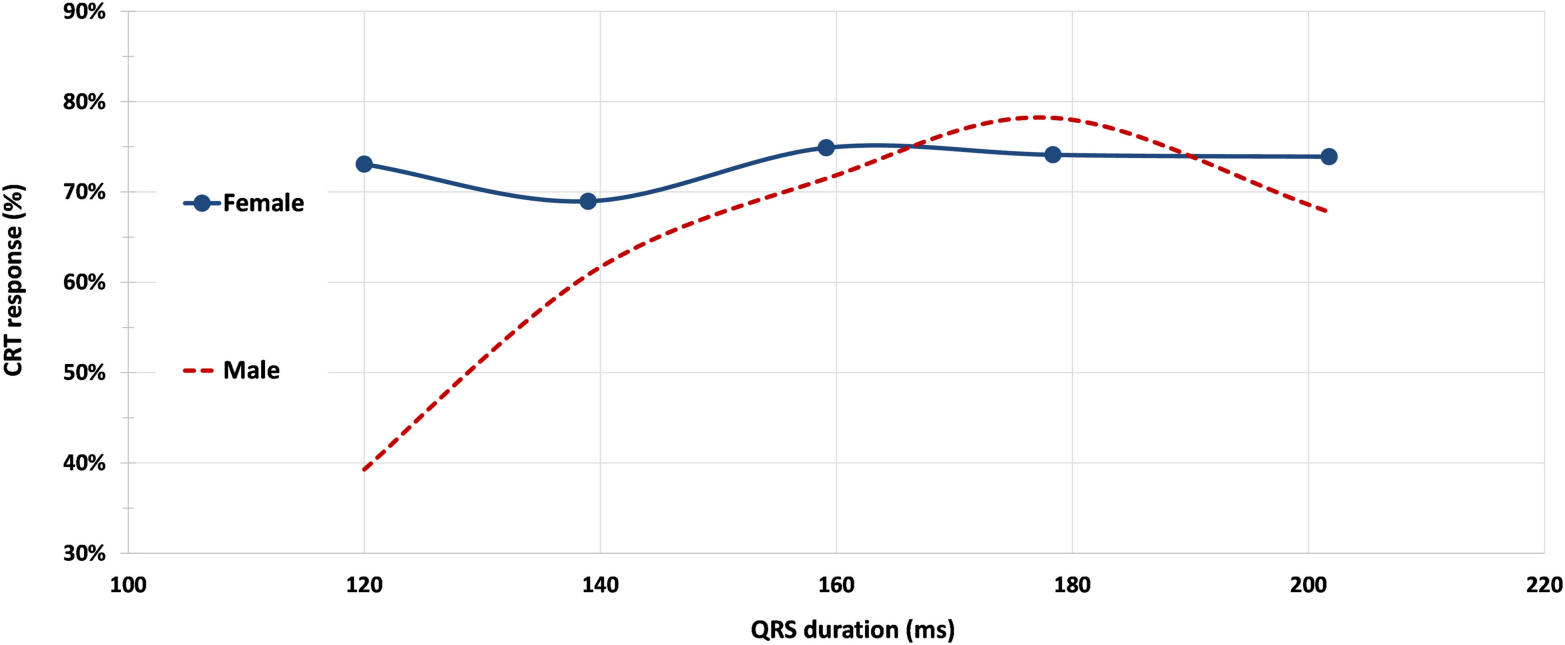
CRT response in patients with NICM and LBBB as a function of QRSd. QRSd was treated as a continuous variable, divided in 5 strata, as follows: strata 1, patients with QRSd <130 ms; strata 2, patients with QRSd ≥130 and <150 ms; strata 3, patients with QRSd ≥150 ms and <170 ms; strata 4, patients with QRSd ≥170 and <190 ms; strata 5, patients with QRSd ≥190 ms. CRT indicates cardiac resynchronization therapy; LBBB, left bundle‐branch block; NICM, nonischemic cardiomyopathy; and QRSd, QRS duration.

**Figure 3:**
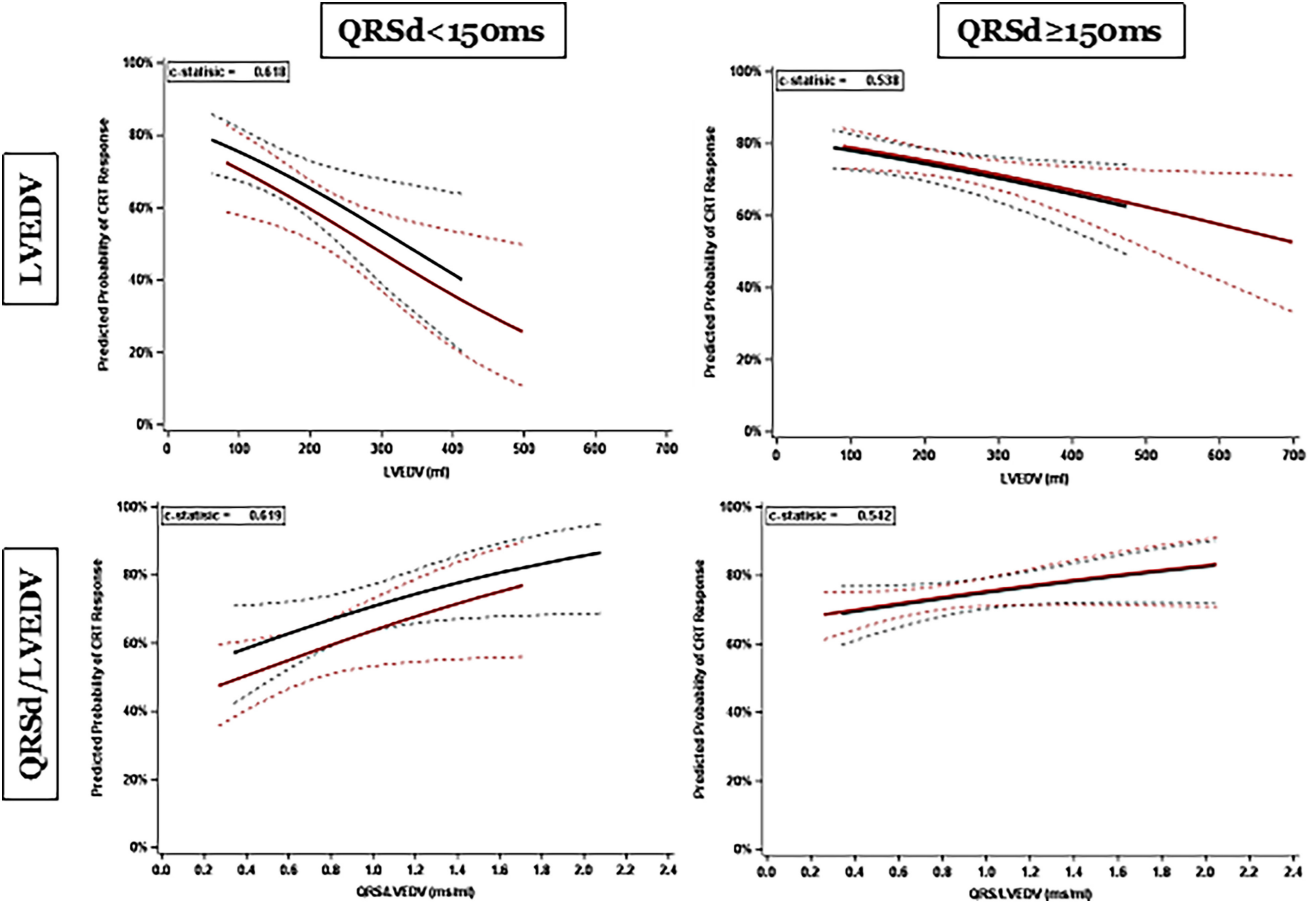
Parametric estimates with corresponding 95% CIs showing predicted CRT response as a function of LVEDV (top 2 parts) and normalized QRSd (bottom 2 parts). The left parts display data for patients with QRSd <150 ms, and the right parts for those with QRSd >150 ms. Blue (with 95% CI): female patients, red (with 95% CI): male patients. CRT indicates cardiac resynchronization therapy; LVEDV, left ventricular end‐diastolic volume; and QRSd, QRS duration. The dashed lines represent the 95% CIs.

In a further analysis of CRT response as a function of QRSd in patients with NICM and LBBB, the female CRT response rate was statistically superior to that of men (69.7% versus 56.9%, *P*=0.015) at QRSd <150 ms (n=315, with 54 patients displaying QRSd <130 ms) (see Figure 2). The predicted probability of CRT response, estimated by the logistic regression model, shows that CRT response improves diminishing LVEDV and increasing QRSd/LVEDV, and that at QRSd <150 ms, female patients have higher CRT response versus male patients (Figure 3). A QRSd/LVEDV of 1.54 in patients with NICM, LBBB, and QRSd <150 ms (area under the curve, 0.62 [95% CI, 0.56–0.69]; *P*=0.0002) was the optimal cut point predicting at least 80% CRT response, based on receiver operating characteristic analysis using the point on the curve closer to the upper left corner (Figure S3). Instead, when considering the entire spectrum of QRS durations, no difference in the predicted probability of CRT response was observed between sexes (Figure S4).

**Figure 4:**
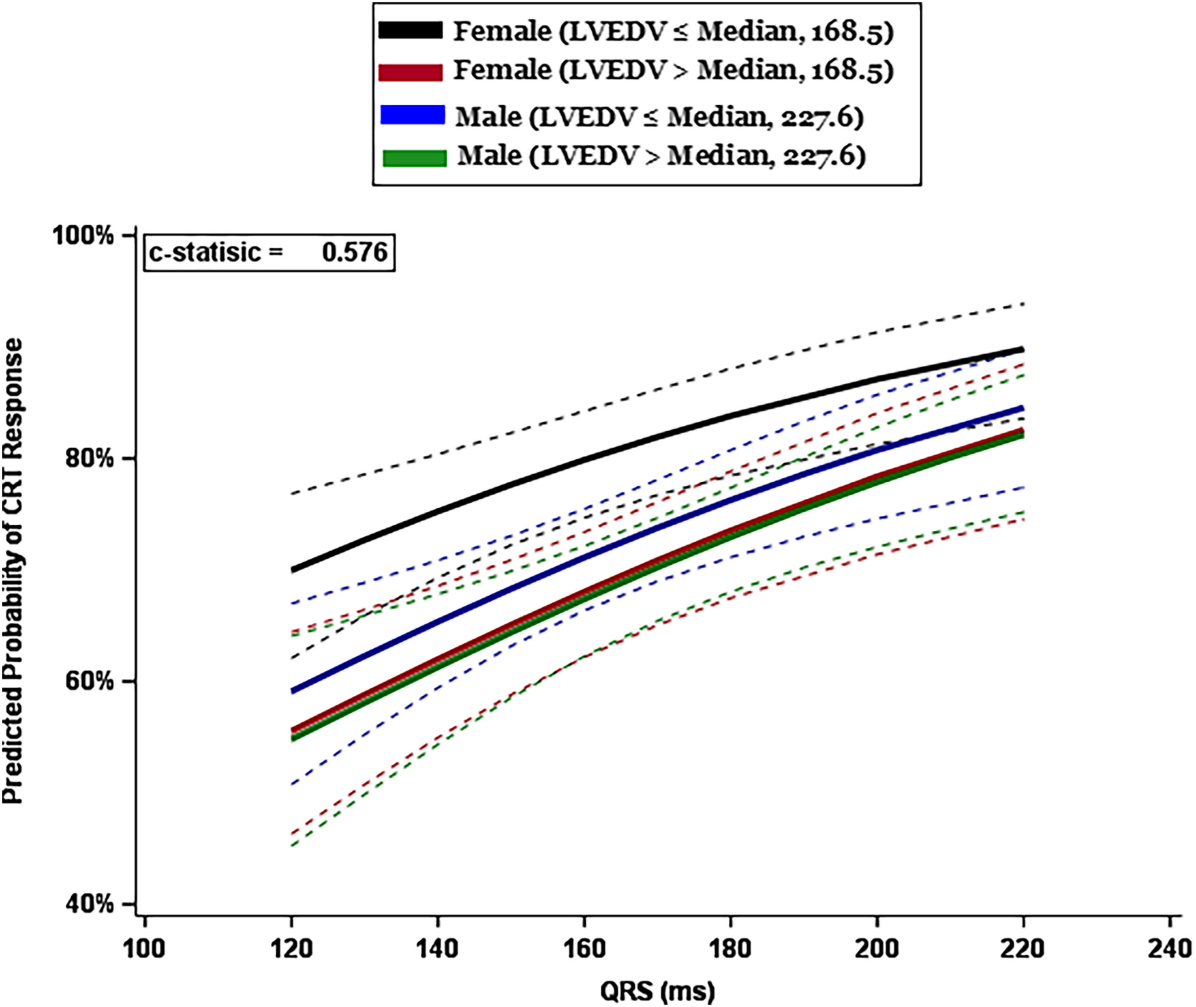
Parametric estimates with corresponding 95% CIs showing predicted CRT response as a function of QRSd in 4 subgroups: women with heart size above and below median LVEDV of 168.5 mL, and men with heart size above and below median LVEDV of 227.6 mL. CRT indicates cardiac resynchronization therapy; LVEDV indicates left ventricular end‐diastolic volume; and QRSd, QRS duration. The dashed lines represent the 95% CIs.

Further parametric estimates are displayed in Figure 4, showing that the cohort with the highest predicted CRT response are female patients with an LVEDV less than the median value (smaller heart size). The difference between male and female patents is not significant at larger heart sizes.

Women with smaller heart size had a significantly higher CRT response than all 3 other cohorts (Table S3): women with a larger heart size (OR, 1.87 [95% CI, 1.26–2.77]); men with a small heart size (OR, 1.61 [95% CI, 1.11–2.33]) and men with a large heart size (OR, 1.92 [95% CI, 1.31–2.82]).

## Discussion

We describe here a post hoc analysis from the prospective multicenter MORE‐CRT MPP, a large trial of 3739 participants, all with complete echocardiographic data available at baseline and 6 months. This large sample size allowed associations between CRT reverse remodeling response and characteristics such as sex to be reliably determined, as well as provided the opportunity to analyze normalized QRS, a baseline variable that is difficult to examine without complete imaging data sets. Importantly, we report results from the largest data set of female patients undergoing CRT ever evaluated to date.^14^ Our findings were as follows:

1. CRT response was superior in women in the overall cohort.
2. In the NICM LBBB cohort, female sex was not an independent response predictor.
3. In the NICM LBBB cohort, increasing normalized QRSd is a significant independent response predictor, with the association between normalized QRSd and response being strongest at QRSd <150 ms.
4. In the NICM LBBB cohort with QRSd <150 ms, CRT response was superior in women.

### Female Sex and CRT Response

In the MORE‐CRT MPP population, CRT response was overall superior among female patients, supporting data from previous studies.^7^, ^8^, ^9^, ^10^, ^11^, ^12^ Our primary logistic regression analysis identified female sex as a significant positive CRT response modifier, as well as the absence of AF, NICM, LBBB, and the absence of renal disease. Interestingly, however, when a subgroup analysis of 1308 patients with NICM and LBBB was performed to control for the effects of cardiomyopathy phenotype and QRS morphology, there was no significant difference in CRT response between female and male patients.

There is conflicting evidence for whether sex is truly an independent modifier of response or whether it is related to confounding variables. Arshad et al^24^ suggested from a post hoc analysis of the MADIT‐CRT trial that sex discrepancy was related to a higher proportion of patients with NICM LBBB in women, factors that are well‐established as being beneficial in CRT response. Although previous studies have reported that female sex may be an independent CRT response modifier, it is important to note the limitations of logistic regression analyses. Use of logistic regression to examine this association may be limited by several factors, such as small female sample size in CRT trials, the assumption of linearity between female sex and CRT response, and the assumption of average or low multicollinearity between dependent and independent variables, such as sex and LVEDV.^25^ Our analysis of a large subgroup of patients with NICM LBBB is consistent with the theory that female sex is not independently associated with CRT response, but rather, is related to confounding from positive response modifiers such as NICM and LBBB.

### Sex‐Specific Impact of QRS, Heart Size, and CRT Response

Our analysis has demonstrated that the sex‐specific divergence in CRT response is most pronounced at shorter QRSd. This supports 2 previous secondary analyses from the MADIT‐CRT (Multicenter Automatic Defibrillator Implantation Trial ‐ Cardiac Resynchronization Therapy) study, which reported that among patients with QRSd <150 ms, only women derived benefit from CRT compared with a implantable cardioverter‐defibrillator.^26^, ^27^

The reasons for this may lie in the discrepancy between male and female heart size, and thus relative dyssynchrony, measured by normalized QRSd. We demonstrate that in the subgroup of patients with NICM LBBB, QRSd/LVEDV is a significant predictor of CRT response. This association is strongest at narrower QRSd and smaller heart sizes. We suggest, therefore, that the sex‐specific differences in CRT response in this subpopulation are a result of increased relative dyssynchrony in the women with narrow QRSd, but reduced heart size compared with their male counterparts, and thus an increased QRSd/LVEDV ratio. This supports previous smaller studies, which have suggested that normalized QRSd may be an appropriate target to guide CRT implantation,^19^, ^20^, ^21^ and mechanistic studies that have reported that echo‐derived dyssynchrony was more predictive of CRT response than absolute QRSd, and may be beneficial in patients with a narrow QRSd.^28^, ^29^ Furthermore, a post hoc analysis of the ECHO‐CRT (Echocardiography Guided Cardiac Resynchronization Therapy)  trial, a study that reported futility in patients with a narrow QRSd implanted with CRT, reported that: (1) men formed the majority of patients and they drove the negative outcome, whereas CRT‐ON versus CRT‐OFF comparison was neutral in women; (2) the higher risk of negative outcome was concentrated among those with larger LV dimensions; and (3) CRT, compared with the control group, induced significant LV reverse remodeling in patients with large normalized QRSd/LVEDV (>1.3 ms/mL).^30^

It should be noted that our results indicate that the strength of the correlation between sex and CRT response appears to be attenuated at high QRSd. We theorize that this is because at these high levels of relative dyssynchrony, CRT response rates are high in both sexes (approximately 70%–75%). Therefore, despite increased QRSd/LVEDV in female patients at these levels, alternative issues are likely to be driving nonresponse, thus diminishing the effect of sex on CRT efficacy at the high extremes of QRSd.

### Clinical Implications

This study demonstrates that heart size is an important factor in driving sex‐specific CRT response, in view of increased normalized QRSd in female patients, with the benefit predominant at narrower QRS durations. These associations were observed through a subgroup analysis of the NICM LBBB cohort that mitigated noise from confounding variables.

Within an NICM LBBB population, selecting CRT recipients based on absolute QRSd dichotomization may exclude certain female patients who could benefit from treatment based on a high degree of relative dyssynchrony due to small heart size. Current European and US guidelines define QRSd cutoffs of 130 and 120 ms, respectively, as the target criteria for CRT implantation.^1^, ^31^, ^32^ These cutoffs are defined from meta‐analyses of studies with few female participants, which may hide a possible beneficial effect of CRT in female patients with narrower QRSd.^33^ This study opens the debate on whether the use of normalized QRSd should be integrated into routine clinical practice to identify these patients. Alternatively, sex‐specific QRSd cutoffs may be considered as a practical surrogate accounting for the significant disparities in normalized QRSd.

## Limitations

This study represents a retrospective analysis of the observational MORE‐CRT MPP trial. As such, we cannot exclude inherent limitations associated with observational studies such as selection bias. In addition, although comprehensive echocardiographic data were gathered, potentially relevant parameters such as LV mass were not collected as part of study protocol. Furthermore, echocardiographic evaluation of response has limitations related to the quality of images obtained, despite this imaging modality being a primary response end point in many historical CRT trials. Nevertheless, the study has important strengths: (1) data collection was prospective, (2) analyses objectives were prespecified, (3) monitoring with strict source data verification activities, (4) echocardiographic evaluations by an echo core laboratory, and (5) the large sample size allowed us to control for confounding patient characteristics, such as ischemic versus nonischemic cardiomyopathy or LBBB versus non‐LBBB QRS morphology. Overall, we are confident that the study data provide a fair description of current CRT application and outcomes; however, our conclusions should be interpreted as hypothesis generating.

## Sources of Funding

None.

## Disclosures

N.W., V.M., F.D.V., and S.H. are supported by the Wellcome/EPSRC Center for Medical Engineering (WT203148/Z/16/Z). N.W. receives fellowship funding from the British Heart Foundation (FS/CRTF/22/24362). S.A.N. acknowledges support from the UK Engineering and Physical Sciences Research Council (EP/M012492/1, NS/A000049/1, and EP/P01268X/1), the British Heart Foundation (PG/15/91/31812, PG/13/37/30280, SP/18/6/33805), US National Institutes of Health (NIH R01‐HL152256), European Research Council (ERC PREDICT‐HF 864055), and Kings Health Partners London National Institute for Health Research Biomedical Research Center. B.T. receives research support from Abbott. M.G. received lecture and consultation fees from Abbott, Medtronic, Boston Scientific, Biotronik, and MicroPort. C.L. receives speaker fees from Medtronic, Abbott, and Biotronik. C.A.R. receives research funding and/or consultation fees from Abbott, Medtronic, Boston Scientific, Spectranetics, EBR Systems, and MicroPort outside of the submitted work. The remaining authors have no disclosures to report.

## Supporting information

Data S1
