# Supplementary material for: Heart Size Difference Drives Sex‐Specific Response to Cardiac Resynchronization Therapy: A Post Hoc Analysis of the MORE‐MPP CRT Trial
Source: J Am Heart Assoc. 2024 Jun 15;13(12):e035279. doi: 10.1161/JAHA.123.035279 (PMC11255746; doi:10.1161/JAHA.123.035279)

SUPPLEMENTAL MATERIAL

Table S1. List of participating institutions and principal investigators in the MORE CRT MPP trial.

Investigational Site	Principal Investigator
Royal Adelaide Hospital	Sanders, Prash
The Alfred Hospital	Mariani, Justin
St. Andrews War Memorial Hospital	Hayes, John
Westmead Hospital	Sivagangabalan, Gopal
Klinik Floridsdorf	Achleitner, Reinhard
Wilhelminenspital Wien	Koch, Johannes
Krankenhaus der Stadt St. Polten	Thudt, Karin
Hopital Erasme	Casado, Ruben
St. Joseph Gilly	Leroy, Jean
Institut de Cardiologie de Montreal (Montreal Heart Inst.)	Thibault, Bernard
Rouge Valley Centenary	Janmohamed, Amir
St. Paul's Hospital	Chakrabarti, Shanta
Institut de Cardiologie de Quebec (Hopital Laval)	Molin, Franck
HSC, Eastern Health	Connors, Sean
QE II Health Sciences	Sapp, John
Kingston General Hospital	Simpson, Christopher
CHUM	Coutu, Benoit
McGill University Health Centre General Hospital	Essebag, Vidal
Royal Alexandra Hospital	Williams, Randall
St. Michael's Hospital	Mangat, Iqwal
Foothills Medical Centre	Sumner, Glen
CHUS Fleurimont	Ayala-Paredes, Felix
Prince of Wales Hospital	Chan, Joseph Yat Sun
Angiografia Clinica de Occidente	Dager Gomez, Antonio
Aalborg Sygehus Syd	Sogaard, Peter
Odense University Hospital	Johansen, Jens Brock
Skejby University Hospital	Nielsen, Jens
Turku University Hospital	Lund, Juha
Keski-Suomi Central Hospital	Nyman, Kai
CHRU Hopital de Pontchaillou	Leclercq, Christophe
CHRU Albert Michallon	Defaye, Pascal
CHRU Lille	Marquie, Christelle
Centre Cardiologique Du Nord	Piot, Olivier
Medipole Lyon-Villeurbanne	Poty, Herve
CHRU de Brest	Mansourati, Jacques
Hopital Clairval	Mechulan, Alexis
Hopital Pitie Salpetriere	Hidden-Lucet, Franciose
Hopital Prive du Confluent	Gras, Daniel
Hopital Saint Philibert	Guyomar, Yves
CHU - Montpellier	Pasquie, Jean-Luc
CHRU Rouen Hospital Charles Nicolle	Anselme, Frederic
CHU de Nancy - Hopital de Brabois	Blangy, Hugues
CHU Hopital G. & R. Laennec	Lande, Gilles
CHR de La Reunion - Site du CHFG	Clerici, Gael
Centre Hospitalier de Belfort-Montbeliard	Fouche, Renaud
Institute Cardio. Paris-Sud - Institut Jacques Cartier	Horvilleur, Jerome
CHRU Hopital de la Cote de Nacre	Pellissier, Arnaud
Hopital Saint Joseph	Gitenay, Edouard

Investigational Site	Principal Investigator
CHU Rangueil Toulouse	Mondoly, Pierre
Centre Hospitalier de Valence	Miralles, Aurelien
CHU du Bocage	Laurent, Gabriel
CHU Gabriel Montpied	Eschalier, Romain
CHU Trousseau	Babuty, Dominique
Deutsches Herzzentrum Munchen des Freistaates Bayern	Kolb, Christof
Medizinische Hochschule Hannover	Veltmann, Christian
Medizinische Einrichtungen der Universitat zu Koln	Steven, Daniel
Berufsgenossenschaftliche Kliniken Bergmannsheil	Boesche, Leif
Herz-und Diabetes Zentrum NRW	Sommer, Philipp
Universitatsklinikum Leipzig AOR	Neef, Martin
Universitatsmedizin Berlin - Campus Benjamin Franklin (CBF)	Huemer, Martin
Evangelisches Krankenhaus Kalk gGmbH	Eberhardt, Frank
Schuchtermann-Schiller'sche Kliniken GmbH & Co. KG	Moennig, Gerold
Universitatsklinikum Munster	Eckardt, Lars
Klinikum Bielefeld gGmbH Klinikum-Mitte	Stellbrink, Christoph
Markische Kliniken GmbH Klinikum Ludenscheid	Lemke, Bernd
Klinikum Ingolstadt GmbH	Seidl, Karlheinz
Helios-Klinikum Erfurt GmbH	Schade, Anja
Klinikum Coburg GmbH	Brachmann, Johannes
Krankenhaus der Barmherzigen Bruder	Voss, Frederik
Universitatsklinikum Greifswald	Busch, Mathias
Herz- und Gefäßzentrum am Krankenhaus Neu-Bethlehem	Hansen, Claudius
Kerckhoff-Klinik gGmbH	Sperzel, Johannes
St.-Marien-Hospital GmbH	Perings, Christian
Kliniken der Friedrich-Alexander-Universitat	Arnold, Martin
Charite Campus Virchow Klinikum	Blaschke, Florian
Hegau-Bodensee-Hochrhein-Kliniken GmbH	Kollum, Marc
Albertinen-Krankenhaus Hamburg	Naegele, Herbert
Stadtisches Klinikum Gutersloh gGmbH	Er, Fikret
St. Vinzenz-Hospital	Winter, Stefan
Universitatsklinikum Wurzburg	Nordbeck, Peter
Universitatsmedizin Gottingen Georg-August-Universitat	Herting, Jonas
Kliniken Villingen-Schwenningen	Jung, Werner
Klinikum St. Georg gGMbH	Klein, Norbert
Elisabeth-Krankenhaus Essen GmbH	Schmitz, Dietmar
Klinikum Oldenburg gGmbH	Oswald, Hanno
Universitats-Herzzentrum Freiburg - Bad Krozingen	Restle, Christian
The Onassis Cardiac Center	Theodorakis, George
Escorts Heart Institute & Research Centre	Saxena, Anil
CARE Banjara	Narasimhan, Calambur
Medanta - The Medicity Hospital	Bhargava, Kartikeya
Care Institute of Medical Sciences	Naik, Ajay
Apollo Hospital	Karthigesan, Arumugam Murugesan
Pushpawati Singhania Hospital & Research Institute	Kler, Tarlochan
Medanta Medicity Hospital	Singh, Balbir
Fortis Hospital	Pal, Shashidhar
Postgraduate Institute of Medical Education & Research	Vijayvergiya, Rajesh
Asian Institute of Gastroenterology (AIG) Hospital	Narasimhan, Calambur
Sheba Medical Center	Bar Lev, David

Investigational Site	Principal Investigator
Rabin Medical Center	Golovchiner, Gregory
Tel Aviv Medical Center	Viskin, Samuel
Hadassah - Ein Kerem	Luria, David
Casa di Cura Dpott. Pederzoli	Vicentini, Alfredo
Policlinico Casilino	Calo, Leonardo
Azienda Ospedaliero Universitaria Pisana	Bongiorni, Maria Grazia
Ospedale Giovanni Paolo II - Cardiology	Nicosia, Antonino
Ospedale Civile Maggiore di Verona Borgo Trento	Morani, Giovanni
Ospedale dei Pellegrini	Ducceschi, Valentino
Azienda Ospedaliera Di Venere	Bonfantino, Vincenzo
Az Osp.Universitaria Maggiore della Carita	Dell'era, Gabriele
Ospedale S. Giovanni Bosco	Capogrosso, Paolo
Ospedale di Portogruaro	Nangah, Rene
Presidio Osp. Vito Fazzi	Pisano, Ennio
Universita degli Studi di Padova	Bertaglia, Emanuele
Casa Sollievo della Sofferenza	Potenza, Domenico Rosario
Az. Osp. Spedali Civili di Brescia	Curnis, Antonio
Az.Osp.Universitaria Consorziale Policlinico	Favale, Stefano
Clinica Mediterranea	Iuliano, Assunta
Azienda Ospedaliera S.Anna e S.Sebastiano	Viscusi, Miguel
Policlinico S.Orsola Malpighi	Ziacchi, Matteo
Az. Osp.Sant'Anna	Russo, Giovanni
Ospedale Madre G. Vannini	Ansalone, Gerardo
Ospedale S. Giovanni Calibita Fatebenefratelli di Roma	Bianchi, Stefano
St. Marianna University School of Medicine Hospital	Harada, Tomoo
American University of Beirut Medical Center	Khoury, Maurice
Institut Jantung Negara	Razali, Omar
Mater Dei Hospital	Aquilina, Oscar
Isala - ZWolle	Delnoy, Peter-Paul
Amsterdam Academic Medical Centre (AMC)	Knops, Reinoud
Catharina Ziekenhuis	Houthuizen, Patrick
TweeSteden Ziekenhuis - Locatie Tilburg	Widdershoven, J.W.M.G.
Medisch Spectrum Twente	Van Es, Jan
Gornoslaskie Centrum Medyczne im.prof. Leszka Gieca	Wilczek, Jacek
Wojewodzki Specjalistyczny Szpital im. Bieganskiego	Bednarkiewicz, Zbigniew
Slaskie Centrum Chorob Serca	Kalarus, Zbigniew
Szpital Kliniczny Przemienienia Panskiego UM w Poznaniu	Mitkowski, Przemyslaw
Santa Maria Hospital	Marques, Pedro
Centro Hospitalar Vila Nova Gaia	Primo, Joao
Hospital de Braga	Rocha, Sergia
Hospital Garcia de Orta, EPE	Brand?o, Luis
Hospital de Santa Cruz	Adragao, Pedro
Centro Hospitalar do Alto Ave, Unidade de Guimarães	Sanfins, Victor
Heart Rhythm Management	Sotomonte Ariza, Juan
Hospital San Lucas Ponce	Perez, Francisco
Meshalkin National Medical Research Center	Romanov, Alexander
King Fahad Medical City	Al Samadi, Faisal
King Fahad Armed Forces Hospital	Bokhari, Fayez
National University Hospital	Seow, Swee Chong
Yonsei University Health System	Joung, Bo Young
Samsung Medical Center	Park, Seung-Jung

Investigational Site	Principal Investigator
Sejong Hospital	Park, Mi Young
Seoul National University Bundang Hospital	Oh, Il Young
Seoul National University Hospital	Oh, Seil
Seoul St. Mary's Hospital	Oh, Yong-Seog
Hospital Universitario Infanta Cristina	Fernandez Concha, Joaquin
Hospital Universitari i Politecnic La Fe	Osca Asensi, Joaquin
Fundacion Jimenez Diaz	Sanchez Borque, Pepa
HCU Virgen de la Victoria	Alzueta Rodriguez, Javier
Hospital Ramon y Cajal	Hernandez Madrid, Antonio
Hospital Alvaro Cunqueiro, Dept of EP & Arrhythmias	Garcia Campo, Enrique
Hospital General Universitario Gregorio Marañon	Arenal, Angel
Hospital Universitario Puerta de Hierro	Toquero-Ramos, Jorge
Hospital de la Santa Creu I Sant Pau	Vinolas, Xavier
Hospital Universitario Doce de Octubre	Lopez-Gil, Maria
Hospital Universitario Virgen de la Nieves	Macias, Maria Rosa
Hospital Universitario A Coruña	Mosquera, Ignacio
Complexo Hospitalario Universitario de Santiago	Martinez Sande, Jose
Hospital Universitario Miguel Servet	Oloriz, Teresa
Karolinska University Hospital, Solna	Gadler, F
Hopital Cantonal Universitaire de Geneva	Burri, Haran
National Taiwan University Hospital	Ho, Li Ting
Chang Gung Memorial Hospital	Wang, Chun-Chieh
John Radcliffe Hospital	Betts, Tim
Golden Jubilee National Hospital	Gardner, Roy
The Royal Sussex County Hospital	Ellery, Sue Mary
Southampton University Hospital	Flett, Andrew
Manchester Heart Center, Manchester Royal Infirmary	Muhyaldeen, Sahrkaw
The Great Western Hospital	Foley, Paul
Queen Elizabeth Hospital	Leyva-Leon, Francisco
St. Thomas Hospital	Rinaldi, Christopher
Kings College Hospital	Scott, Paul
Wansbeck General Hospital	Runnett, Craig
Cardiac Rhythm Specialists, Inc.	Polosajian, Leo
Heart Center Research, LLC.	Jennings, John
Lancaster General Hospital	Bansal, Sandeep
Baptist Health Lexington	Tomassoni, Gery
Redmond Regional Medical Center	Styperek, Robert
Cardiovascular Associates of Mesa	Kaplan, Andrew
Colorado Heart & Vascular, P.C.	Venkataraman, Ganesh
Samaritan Heart & Vascular Institute - Cardiology Dept.	Hsing, Jeff
Glendale Adventist Medical Center	Mckenzie, John
St. Francis Hospital	Sellers, Matthew
Comprehensive Cardiovascular	Habib, Moksedul
EP Heart	Hariharan, Ramesh
Erlanger Medical Center	Manyam, Harish
Phoenix Cardiovascular Research Group	Bahu, Marwan
Baylor All Saints Medical Center at Fort Worth	Shah, Syed
Central Cardiology	Salvo, Jared
McLaren Health Care Corporation	Buerkel, Daniel
Memorial Katy Cardiology Associates	Kashani, Amir
Deborah Heart & Lung Center	Corbisiero, Raffaele

Investigational Site	Principal Investigator
Scripps Health	Rogers, John
Vivek Mangla, MD	Mangla, Vivek
Coliseum Medical Centers	Hoffman, Jonathan
MidMichigan Medical Center-Midland	Islam, Nilofar
San Diego Cardiac Center	Athill, Charles
Brigham & Women's Hospital	Koplan, Bruce
Cardiology Associates of Fairfield County, PC	Tiano, Joseph
Cardiovascular Associates of the Delaware Valley	Levi, Steven
Eisenhower Medical Center	Feldman, Leon
Methodist University Hospital	Jha, Sunil
Sansum Clinic - Santa Barbara Medical Foundation	Cogert, Gregory
St. Elizabeth Medical Center - South Unit	Sinno, Mohamad
Munson Medical Center	Jaffe, Brian
CHI Health Creighton University Medical Center-Bergan Mercy	Abuissa, Hussam
St. Vincent Heart Clinic Arkansas	Chakka, Mangaraju

Table S2. Sensitivity analysis. Multivariable regression table of the NICM LBBB population excluding diabetes and AF, which were significant predictors in the full multivariable regression analysis.

	Odds ratio		
Parameters	Point estimate	95% confidence interval	P vale
Age	0.999	0.988-1.011	
QRSd/LVEDV	1.955	1.230-3.107	0.005
Female vs Male	1.003	0.772-1.304	

Table S3. Head-to-head comparison of CRT response rates in females with small heart sizes (<median value of 168.5ml, FS), females with large heart sizes (>median value, FL), males with small heart sizes (<median value 227.6ml, MS) and males with large heart sizes (>median value, ML). Results expressed as odds ratio, OR, with 95% confidence intervals.

Comparison	Odds Ratio [95% confidence interval]
FS versus FL	1.87 [1.26, 2.77]
FS versus MS	1.61 [1.11, 2.33]
FS versus ML	1.92 [1.31, 2.82]
MS versus ML	1.19 [0.87, 1.63]
FL versus ML	1.03 [0.73, 1.45]

Figure S1. CONSORT diagram.

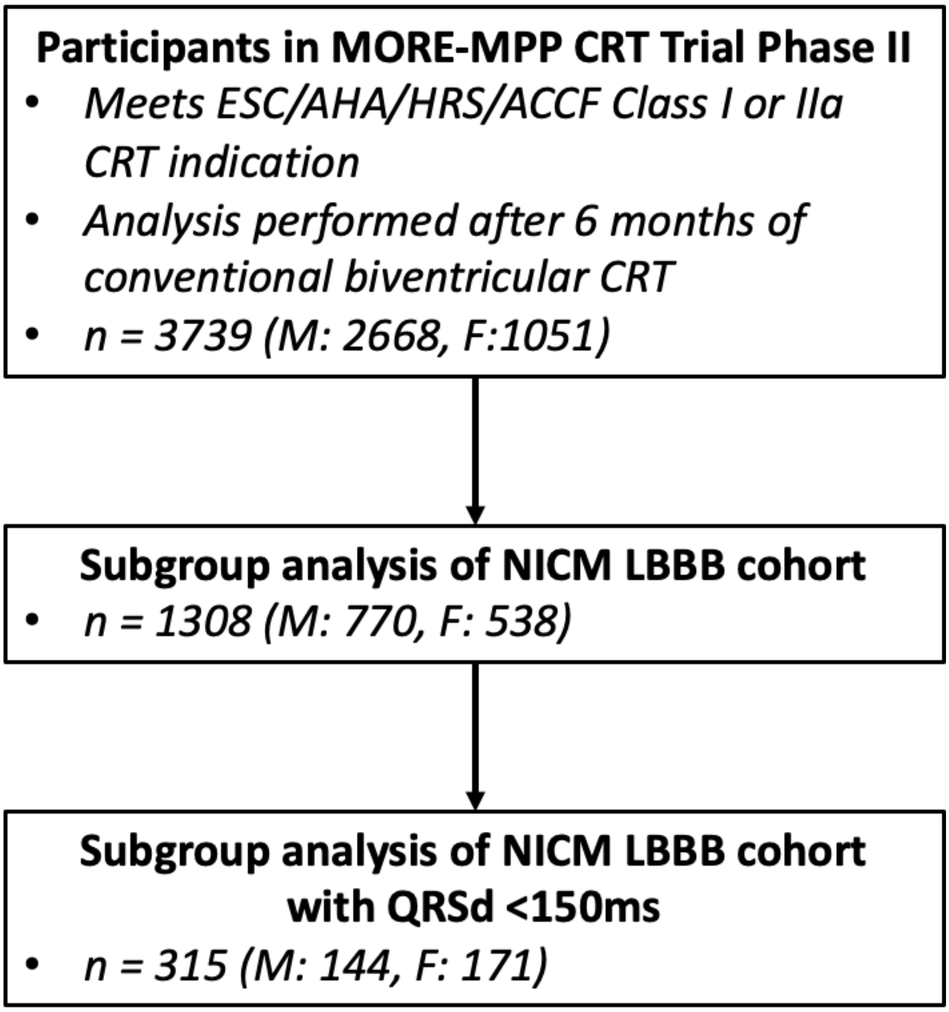

Figure S2. CRT response as function of QRSd/LVEDV in NICM LBBB male patients. QRSd/LVEDV was treated as a continuous variable and divided into 10 deciles.

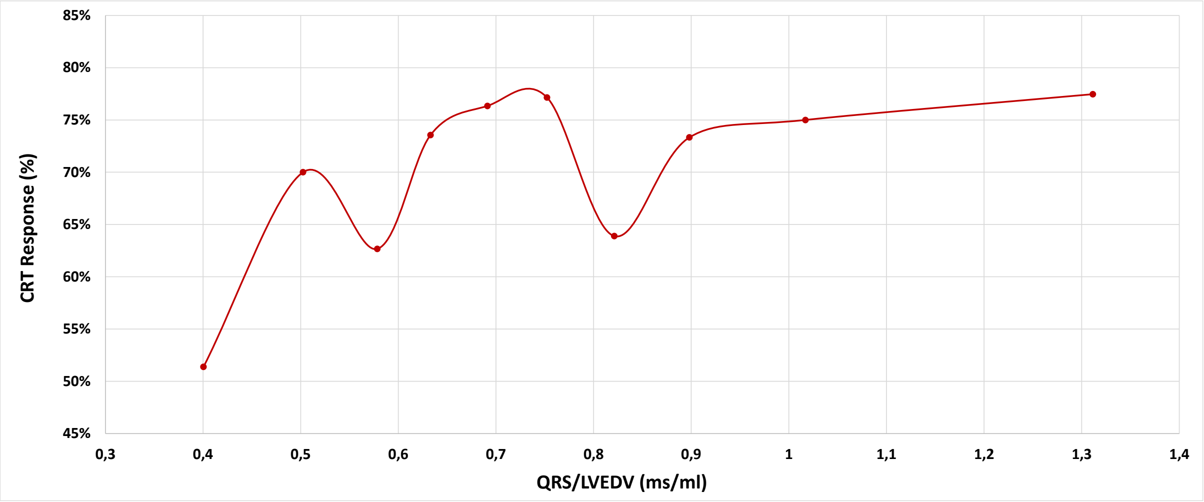

Figure S3. ROC analysis of CRT response as function of QRSd/LVEDV in NICM, LBBB, QRSd<150ms cohort.

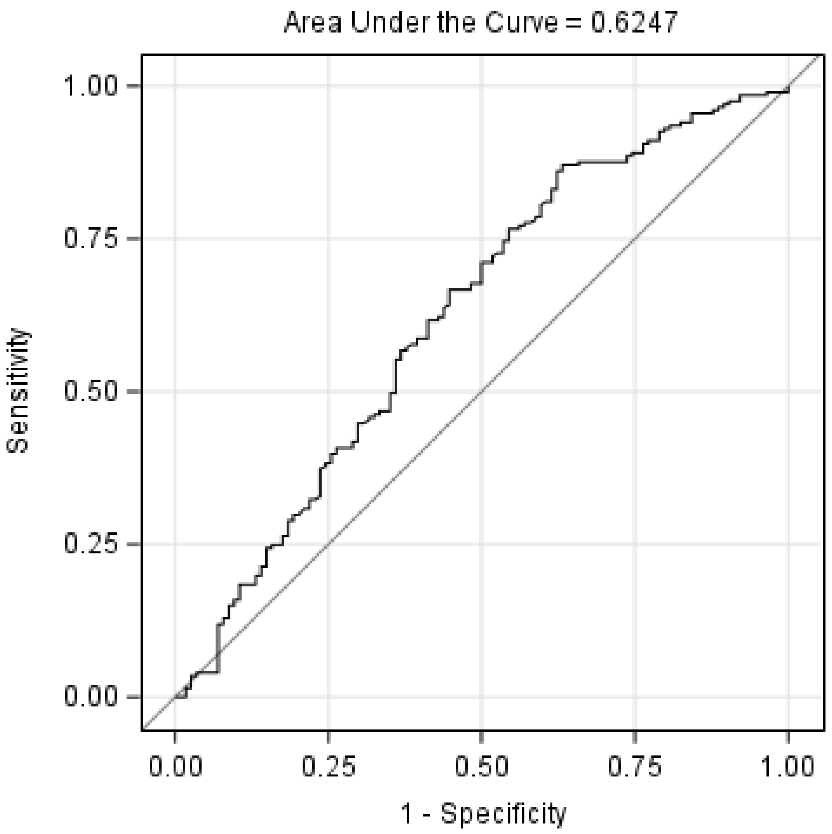

Figure S4. Parametric estimates with corresponding 95% confidence intervals showing predicted CRT response as function of LVEDV (A) and QRSd/LVEDV (B). Blue (with 95% CI): female patients, red (with 95% CI): male patients.

A.

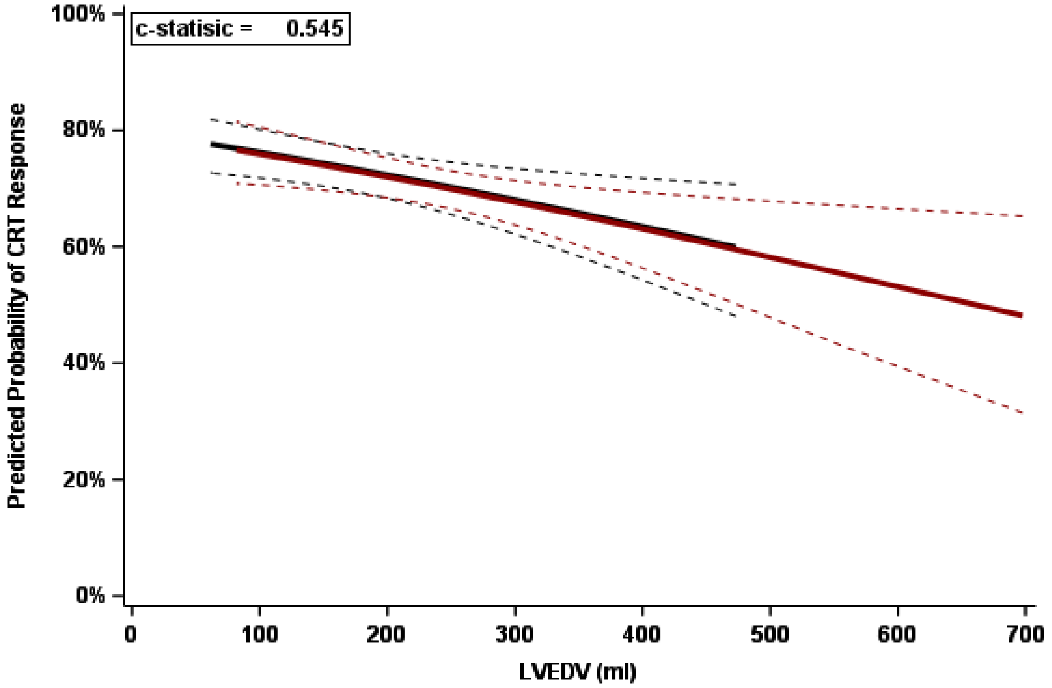

B.

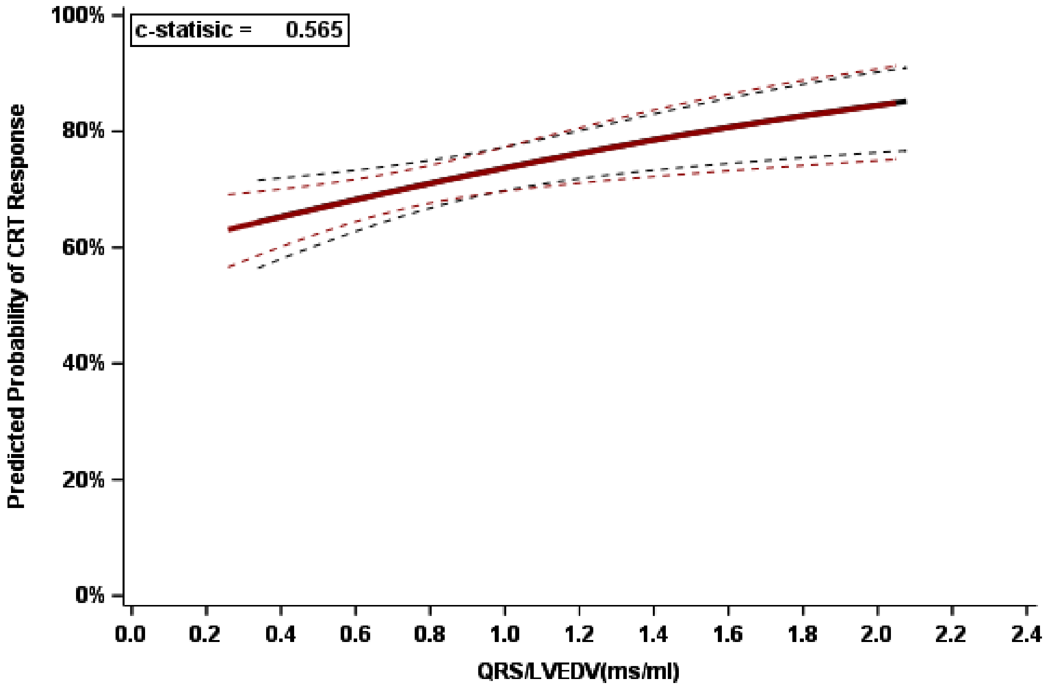

Supplement: Supplementary file 1 — Data S1 [file JAH3-13-e035279-s001.pdf]
